# Molecular Typing and Resistance Profile of *Acinetobacter baumannii* Isolates during the COVID-19 Pandemic: Findings from the “EPIRADIOCLINF” Project

**DOI:** 10.3390/antibiotics12101551

**Published:** 2023-10-19

**Authors:** Antonella Agodi, Arturo Montineri, Rosa Manuele, Paola Noto, Giuseppe Carpinteri, Giacomo Castiglione, Patrizia Grassi, Antonio Lazzara, Anna Rita Mattaliano, Giuseppa Granvillano, Claudia La Mastra, Maria Clara La Rosa, Andrea Maugeri, Martina Barchitta

**Affiliations:** 1 Department of Medical and Surgical Sciences and Advanced Technologies “GF Ingrassia”, University of Catania, 95123 Catania, Italy; claudia.lamastra@unict.it (C.L.M.); mariaclara.larosa@unict.it (M.C.L.R.); andrea.maugeri@unict.it (A.M.); martina.barchitta@unict.it (M.B.); 2 Azienda Ospedaliero-Universitaria Policlinico AOUP “G. Rodolico-San Marco”, 95123 Catania, Italy; a.montineri@libero.it (A.M.); rosimanuele@gmail.com (R.M.); giacomo.castiglione@tin.it (G.C.); antonio.lazzara@alice.it (A.L.); anna.matar@gmail.com (A.R.M.);

**Keywords:** SARS-CoV-2, healthcare-associated infection, AMR, clonal relationships

## Abstract

Due to the COVID-19 pandemic, there has been a shift in focus towards controlling the spread of SARS-CoV-2, which has resulted in the neglect of traditional programs aimed at preventing healthcare-associated infections and combating antimicrobial resistance. The present work aims to characterize the colonization or infection with *Acinetobacter baumannii* of COVID-19 patients and to identify any clonality between different isolates. Specifically, data and resistance profiles of *A. baumannii* isolates were prospectively collected from patients recruited by the EPIRADIOCLINF project. Pulsed-field gel electrophoresis (PFGE) and multi-locus sequence typing (MLST) were used for molecular typing. Overall, we analyzed 64 isolates of *A. baumannii* from 48 COVID-19 patients. According to our analysis, we have identified the spread of a clonally related isolate, referred to as B. The PFGE pattern B includes four subtypes: B1 (consisting of 37 strains), B2 (11), B3 (5), and B4 (2). Furthermore, in the isolates that were examined using MLST, the most observed sequence type was ST/281. In terms of resistance profiles, 59 out of the total isolates (92.2%) were found to be resistant to gentamicin, carbapenems, ciprofloxacin, and tobramycin. The isolation and identification of *A. baumannii* from COVID-19 patients, along with the high levels of transmission observed within the hospital setting, highlight the urgent need for the implementation of effective prevention and containment strategies.

## 1. Introduction

Antimicrobial resistance (AMR) poses a significant public health problem globally, given its substantial clinical and economic impact [1,2,3]. Recent estimates from the European Union/European Economic Area (EU/EEA) indicate that over 670,000 infections and nearly 33,000 deaths per year are caused by AMR pathogens [4]. Although antibiotic misuse is the primary contributor to AMR, other factors play an important role in determining the spread of AMR pathogens and genetic elements of resistance in humans, animals, and the environment [5]. Additional contributors include poor infection control practices, a lack of access to clean water, sanitation, and hygiene, abuse of antibiotics in agriculture and food production, international travel and trade, and horizontal gene transfer in bacteria [6,7,8]. More recently, some studies have also shown that certain factors (such as social, economic, organizational, educational, and environmental factors) can account for differences between countries at a global and European level [5,9,10,11,12,13,14]. Italy has one of the highest levels of AMR compared with other European countries, as confirmed by a country visit from the European Center for Disease Prevention and Control (ECDC) in 2017 [15]. Specifically, during this country visit, high levels of carbapenem-resistant Enterobacteriaceae (CRE) and *Acinetobacter baumannii*, as well as methicillin-resistant *Staphylococcus aureus* (MRSA), were reported [15]. A comparable scenario has been noted on a regional scale, especially within Sicily [16,17,18], the Mediterranean’s largest island and an autonomous region in Southern Italy. Sicily has a population of nearly 5 million people, around 51% of which are women and 50% are over the age of 45. In 2019, there were 67 public hospitals and 59 accredited healthcare facilities in Sicily, with a total of 3.1 beds per 1000 inhabitants, according to the latest available data [19]. 

Strategies to reduce this risk of AMR include the improvement of clinical management, the prevention of transmission in all healthcare settings, and the prevention of cross-border transmission. Other options include timely laboratory reporting, screening and isolation of patients at higher risk, good infection prevention and control (IPC) measures, effective cleaning and disinfection procedures, and implementation of antimicrobial stewardship programs [20]. To address the AMR issue, in 2017, Italy adopted its first National Action Plan on Antimicrobial Resistance (Piano Nazionale per il Contrasto dell’Antimicrobico Resistenza, PNCAR 2017–2020) [21]. The PNCAR 2017–2020 was a comprehensive framework to contrast AMR, with specific objectives and actions outlined for both human and animal fields. It utilized a One Health strategy, leveraging the synergy between national, regional, and local levels [21]. However, Italy has a decentralized healthcare system in which regional administrations play an important role in IPC strategies. For this reason, the Health Authority of the Sicilian Region implemented a Regional Action Plan for healthcare-associated infection (HAI) and AMR prevention, which included a surveillance system for monitoring, among other things, AMR and antibiotic consumption at hospital and community level [22]. The ultimate goal of the regional surveillance system was to improve the evaluation of the effectiveness of programs aimed at reducing antibiotic misuse and the impact of AMR [22]. 

Within this context, *A*. *baumannii* warrants specific consideration. Although it is one of the less commonly reported bacterial species in the EU/EEA, as per data from the European Antimicrobial Resistance Surveillance Network (EARS-Net), it also exhibits one of the most significant inter-country variations in the prevalence of AMR [4]. In fact, the percentage of isolates resistant to at least one antimicrobial group under surveillance (i.e., fluoroquinolones, aminoglycosides, or carbapenems) varied significantly in 2020, ranging from 0.0% to 98.2%. The highest values were reported in countries located in southern and eastern Europe [4]. *A*. *baumannii* isolates resistant to at least three different class of antimicrobials (such as cephalosporins, penicillins, combinations of β-lactam and β-lactamase inhibitors, monobactam, fluoroquinolones, and aminoglycosides) are generally defined as multidrug-resistant (MDR) [23]. Within healthcare settings, MDR *Acinetobacter* spp. poses a significant threat as it is notoriously challenging to eliminate once it becomes established [24]. According to leading international organizations, MDR poses a significant concern for *Acinetobacter* spp.—particularly in countries where AMR is more prevalent [25]—as it restricts treatment options for patients. In particular, the World Health Organization (WHO) has included carbapenem-resistant *A*. *baumannii* on its global priority list of AMR bacteria [26], while the ECDC has encouraged further efforts to prevent the spread of carbapenem-resistant *A*. *baumannii* in all healthcare settings [27].

To this already complex scenario, the potential impact of the COVID-19 pandemic has been added. Since the onset of the COVID-19 pandemic in 2020, there has been a special focus on controlling the spread of SARS-CoV-2 infection. This has led to a neglect of traditional programs aimed at preventing HAIs and AMR [28,29,30]. Coinfection and secondary infections in COVID-19 patients have been reported globally, with a range of 0.6% to 45% [31]. Recent studies indicate that bacterial coinfection at admission was found in 3.1% to 3.5% of COVID-19 patients, while secondary bacterial infections following hospitalization occurred in up to 15% of patients [31,32,33]. A study conducted in Wuhan (China) showed that *A*. *baumannii* was the predominant bacterial secondary infection, with 91.2% of isolates exhibiting resistance to carbapenem [34]. Patients who are infected or colonized with *A*. *baumannii* can act as a significant source for the horizontal transmission in all healthcare facilities [16,35,36,37]. Moreover, MDR *A*. *baumannii* infections were found to be among the most prevalent bacterial co-infections in COVID-19 patients [38,39]. From a clinical perspective, infections caused by *A*. *baumannii*, especially those caused by MDR strains, were among the leading causes of severe disease and poor prognosis in COVID-19 patients [40,41,42]. 

To address this issue, we launched the EPIRADIOCLINF project in 2020, with the overarching goal of developing an integrated approach to evaluate the impact of epidemiological, radiological, clinical, and molecular characteristics on the diagnosis and management of COVID-19. The project, which is still ongoing, focuses on COVID-19 patients who have been admitted to the Azienda Ospedaliero Universitaria “G. Rodolico-San Marco” in Catania (Sicily, Italy). Within the framework of this comprehensive project, the present study aimed to investigate the colonization and/or infection status of *A*. *baumannii* among COVID-19 patients who were enrolled between August 2020 and April 2021. Specifically, we conducted molecular typing and tested resistance profiles of *A*. *baumannii* in order to identify any possible clonality between different isolates. 

## 2. Results

### 2.1. Study Population

The present analysis was conducted on 64 *A*. *baumannii* isolates that were prospectively collected from 48 COVID-19 patients (resulting in an average of 1.3 isolates per patient) of the Azienda Ospedaliero Universitaria “G. Rodolico-San Marco” of Catania (Italy). Specifically, these patients were admitted to three different hospital wards from August 2020 to April 2021: 36 (75%) patients were admitted to the intensive care unit (ICU), 10 (20.8%) to Infectious and Tropical Diseases, and 2 (4.2%) to the Emergency ward. Overall, patients were aged from 39 to 90 years, with a median age of 70 years, and approximately 71% of patients were male. Regarding pre-existing comorbidities, 65.7% of patients had hypertension, 44.4% had diabetes, and 37.1% had ischemic heart disease. Regarding COVID-19, 52.1% exhibited a severe form of the disease and 64.6% died in the hospital. 

#### Antimicrobial Treatment

Out of the 48 patients included in the current analysis, details regarding their antibiotic treatment were accessible for 40 individuals (Table 1). Antimicrobial drugs for systemic use, categorized under the Anatomical Therapeutic Chemical (ATC) class J01CR, which encompasses combinations of penicillins with beta-lactamase inhibitors, constituted 52.5% of the prescribed antimicrobial agents. Specifically, piperacillin and beta-lactamase inhibitor (J01CR05) represented 50% of these agents, while amoxicillin and beta-lactamase inhibitor (J01CR02) accounted for 2.5%. Third generation cephalosporins (ATC class J01DD), particularly ceftriaxone (J01DD04), represented 17.5%. Other antimicrobial groups were prescribed infrequently, including macrolides (clarithromycin, ATC class J01FA09, 10%), polymyxins (colistin, ATC class J01XB01, 5%), carbapenems (meropenem, ATC class J01DH02, 5%), glycopeptides (teicoplanin, ATC class J01XA02, 2.5%), tetracyclines (tigecycline, ATC class J01AA12, 2.5%), and other antibacterials (linezolid, ATC class J01XX08, 5%). In addition, 36 patients were receiving therapy with at least two antimicrobial agents, with carbapenems being the most commonly administered antimicrobial agents, accounting for 22.2% of cases. Table 2 and Table 3 show the results among patients who received at least two or three antimicrobial agents, respectively. 

### 2.2. Characteristics of Isolates

**Figure 1:**
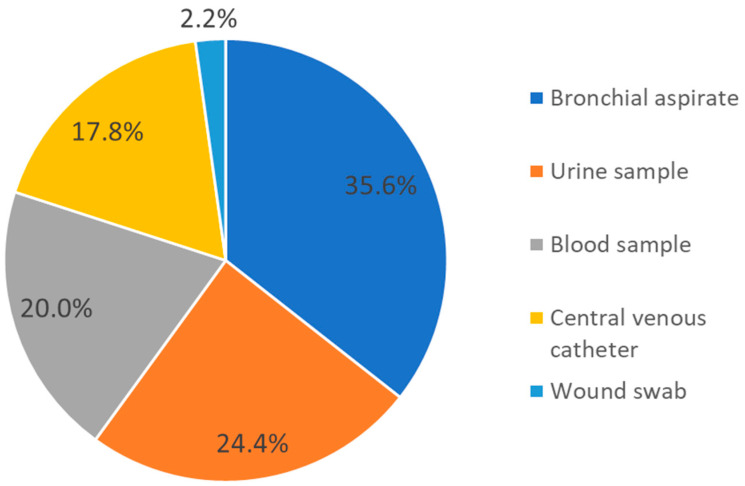
Distribution of samples from which *A. baumannii* was isolated and associated with patient colonization/infection.

Among the 64 A. baumannii isolates, 45 (accounting for 70.3% of the total isolates) were linked to patient colonization or infection. Among these isolates, A. baumannii was detected in 16 bronchial aspirates (35.6%), 11 urine samples (24.4%), 9 blood samples (20%), 8 central venous catheters (17.8%), and 1 wound swab (2.2%) (Figure 1). The remaining 19 isolates (29.7% of the total isolates) were identified as carriage.

#### 2.2.1. Resistance Profiles

Regarding the resistance profiles of isolates linked to colonization or infection, 59 out of 64 (92.2%) were resistant to gentamicin, carbapenems, ciprofloxacin, and tobramycin. Only one isolate was susceptible to the mentioned antibiotic classes, while the remaining four were not tested. Additionally, 53 out of 64 (82.8%) isolates were resistant to amikacin, while seven were not tested, and 56 out of 64 (87.5%) isolates were resistant to trimethoprim with sulfamethoxazole, while four were not tested.

#### 2.2.2. Clonal Relationships

**Figure 2:**
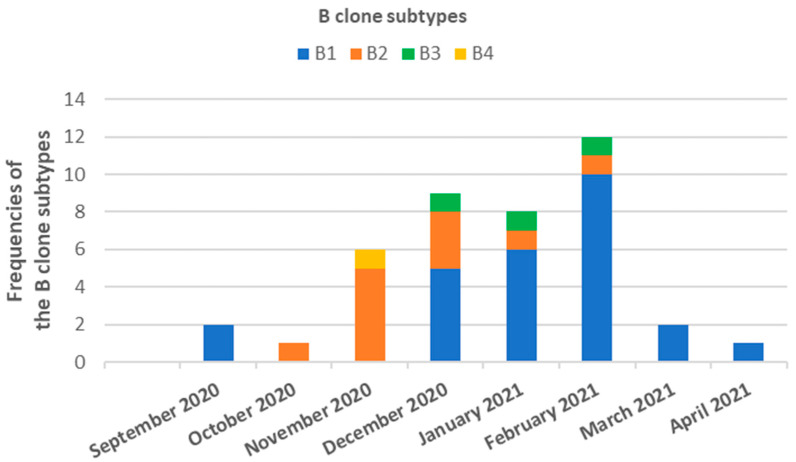
Temporal distribution and frequency of clone B of *A. baumannii* strains isolated from COVID-19 patients admitted in ICU, Infectious and Tropical Diseases or Emergency wards of the Azienda Ospedaliero Universitaria “G. Rodolico-San Marco” of Catania (Sicily, Italy) between September 2020 and April 2021.

The analysis revealed eight distinct pulsed-field gel electrophoresis (PFGE) patterns, labeled A–H, which were not related to each other. With the exception of patterns A, B, and F, all remaining patterns were associated with sporadic strains. Patterns A and F were detected in two isolates, both originating from the same patient. Pattern B was detected in 55 isolates (86.0% of all isolates) and collected from 41 patients (85.4% of all patients). Out of the entire set of B clones, 39 strains (71% of all B clones) were obtained from patients who were hospitalized in the ICU, 14 (25.4%) from the Infectious and Tropical Diseases ward, and 2 (3.6%) from the Emergency ward. The B clone comprised 38 (69.1%) colonization or infection cases, where the bacterium was isolated from bronchial aspirate samples (*n* = 15; 27.3%), blood samples (*n* = 8; 14.5%), central venous catheter (*n* = 8; 14.5%), urine samples (*n* = 6; 10.9%), and wound swab (*n* = 1; 1.8%), while the remaining isolates were carriage. Pattern B was further classified into subtypes: B1 (37 strains), B2 (11 strains), B3 (5 strains), and B4 (2 strains). Subtypes B1, B2, and B3 were predominantly found in patients admitted to the ICU and the Infectious and Tropical Diseases ward, with subtype B3 also being detected in the Emergency ward. Subtype B4, on the other hand, was exclusively isolated in the Infectious and Tropical Diseases ward. In terms of temporal patterns, subtype B1 was initially isolated in September 2020, experienced a gap, and then reappeared in November 2020, continuing to be isolated until April 2021. Subtypes B2 and B3 were identified from October 2020 to February 2021 and from December 2020 to February 2021, respectively. Subtype B4, however, was only detected in November 2020 and was not encountered again (Figure 2).

#### 2.2.3. Molecular Typing by Multi-Locus Sequence Typing

Eleven isolates, exhibiting diverse PFGE patterns (A–H) and representing subtypes B1, B2, B3, and B4, were randomly chosen and subjected to multi-locus sequence typing (MLST) analysis. The following seven internal housekeeping genes were sequenced: citrate synthase (gltA), DNA gyrase subunit B (gyrB), glucose dehydrogenase B (gdhB), homologous recombination factor (recA), 60-kDa chaperonin (cpn60), glucose-6-phosphate isomerase (gpi), and RNA polymerase sigma factor (rpoD). Using the Oxford scheme, five sequence types (STs) were assigned to 11 different PFGE profiles, including sub-types B1, B2, B3, and B4. The most common sequence type was ST/281, followed by ST/238. Among the eleven isolates, six belonged to ST/281, two to ST/238, and one each to ST/978, ST/782, and ST/1893 (Table 4).

## 3. Discussion

To our knowledge, this is the first study reporting an analysis of *A*. *baumannii* clonality among COVID-19 patients in Sicily (Italy). As previously highlighted in the introduction, tackling the challenge of AMR demands regional initiatives, particularly in countries like Italy, which have a decentralized healthcare system. Specifically, Sicily bore a substantial burden during the COVID-19 pandemic. The initial confirmed case of COVID-19 in Sicily was recorded in late February 2020, swiftly transforming the region into one of the most severely affected areas in the country. During the pandemic, Sicily has enacted several measures in an attempt to manage the virus’s transmission. These measures encompassed lockdowns, travel limitations, and compulsory mask wearing in public spaces [43]. Despite significant progress in managing the pandemic globally, it has had notable effects on IPC practices as well as on AMR. Firstly, the increased demand for healthcare services and the need to prioritize COVID-19 patients has resulted in a reduced focus on other HAIs, leading to a potential increase in their incidence [28,44,45]. Secondly, the extensive and often inappropriate use of antimicrobial agents in COVID-19 patients has led to an increase in the prevalence of AMR [28,44]. Furthermore, the pandemic has also disrupted supply chains and global health systems, resulting in limited access to essential drugs and diagnostic tools, which can contribute to the worsening of AMR [28,44,45]. Finally, the implementation of IPC measures, such as social distancing and reduced contact, has resulted in a decreased incidence of some infections, while exacerbating the risks of other infections and increasing the need for appropriate prevention measures [28,44,45]. More directly, COVID-19 patients are frequently subjected to intensive care and mechanical ventilation [46,47,48,49,50,51,52,53,54]. Hospitalization rates for COVID-19 patients range from 5% to 15%, with some requiring ICU follow-up [44]. Recent reports also suggest that up to 80% of ICU-admitted COVID-19 patients require invasive mechanical ventilation [55]. Certain risk factors, such as invasive and non-invasive mechanical ventilation, industrial oxygen administration, invasive procedures, and prolonged hospital stay, were associated with increased incidence of HAIs infection during the COVID-19 pandemic [56]. Not in the least, it is known that COVID-19 patients often receive a high amount of antibiotics. 

While all patients are susceptible to HAI, COVID-19 patients may be even more vulnerable, and any co-infection can exacerbate their clinical presentation and prognosis [57]. Irrespective of the pandemic situation, infections caused by *A*. *baumannii*—and particularly those sustained by MDR strains—result in a higher risk of adverse outcomes, prolonged hospitalization, and increased costs [3,58,59,60]. In fact, *A*. *baumannii* has developed various mechanisms of resistance, such as acquiring β-lactamases, up-regulating multidrug efflux pumps, modifying aminoglycosides, developing permeability defects, and altering target sites [61]. These mechanisms of resistance primarily involve regulating the transport of antibiotics through bacterial membranes, altering the antibiotic target site, and enzymatically modifying antibiotics to neutralize their effects [61]. For this reason, among the numerous challenges posed by the pandemic, it is particularly important to highlight the occurrence of multiple outbreaks of MDR and extensively drug-resistant (XDR) *A*. *baumannii* in healthcare settings [62,63]. MDR and XDR strains of *A*. *baumannii* have been frequently identified as causative agents of secondary bacterial infections in COVID-19 patients, highlighting the urgent need for effective IPC measures [64,65]. During the pandemic, in fact, the incidence of infections caused by MDR *A*. *baumannii* was higher than in the pre-pandemic period [66]. The high incidence of secondary infections caused by MDR *A*. *baumannii* has been also associated with prolonged ICU stay, mechanical ventilation, and higher mortality rates [67]. Infections caused by MDR *A*. *baumannii* worsen the prognosis of critical and immunocompromised COVID-19 patients, significantly increasing morbidity and mortality rates [40,41,42]. Specifically, secondary bacterial infection with MDR *A*. *baumannii* led to a two-fold increase in COVID-19-related mortality [64]. 

Our initial observation revealed that over half of patients were undergoing treatment involving a combination of penicillins, including beta-lactamase inhibitors. Furthermore, nearly all patients were receiving therapy involving at least two antimicrobial agents, with carbapenems being the most commonly prescribed among them. It is worth noting that the utilization of carbapenems has the potential to disrupt the natural bacterial microbiota in patients, potentially elevating the risk of colonization and/or infection by AMR bacteria [68,69]. At the molecular level, we also detected a prevailing PFGE pattern, which was evident in the majority of isolates and COVID-19 patients, with prevalence rates exceeding 85% in both instances. Furthermore, it was feasible to categorize this PFGE pattern into four subtypes (B1, B2, B3, and B4), each exhibiting different distributions across hospital wards and throughout the study period. Specifically, our study identified the B1 and B2 subtypes for a consecutive period of 5 and 4 months, respectively. The isolation of these subtypes in multiple wards suggests a potential spread that may have been indirectly mediated through contaminated environments and healthcare personnel. Our findings align partially with those of Ceparano and colleagues, who conducted a study characterizing the clonal spread of *A*. *baumannii* among COVID-19 patients admitted to an ICU in Lazio, Italy. They reported a cumulative incidence of *A*. *baumannii* colonization or infection at 36.8%, and patients with *A*. *baumannii* had increased mortality rates and longer hospital stays. Notably, previous carbapenem usage emerged as the primary risk factor linked to *A*. *baumannii* acquisition. Additionally, the authors documented significant instances of *A*. *baumannii* infections and colonization, along with notable levels of clonal transmission [70]. Other studies have already suggested evidence of possible indirect transmission, resulting in epidemic outbreaks that persist for an extended period of time [71,72]. Thoma and colleagues [73] performed a systematic review of MDR outbreaks, including those caused by carbapenem-resistant *A*. *baumannii* (CRAB), during the COVID-19 pandemic, and identified the factors that contributed to these outbreaks. The study revealed that the most common contributing factor was an increase in patient mobility due to hospital overcrowding and the need to transfer patients to different locations [73]. Other factors included a lack of personal protective equipment and understaffing due to healthcare worker infections or redeployment to COVID-19 units. These factors increased the risk of transmission of MDR bacteria, including CRAB [73].

The surveillance of *A*. *baumannii* through molecular characterization is essential not only for outbreak control, but also in the context of AMR. To investigate this question, several studies have used MLST and whole-genome sequencing (WGS). A study conducted by Wareth et al. in Southeast Asia uncovered a broad spectrum of antimicrobial resistance (AMR) genes responsible for resistance to various antimicrobial agents. This research revealed a high degree of diversity among the strains through the utilization of Next Generation Sequencing (NGS) technology [74]. Another study by Hammerum et al. in Denmark examined a potential outbreak of CRAB and conducted a comparative analysis using three distinct typing methods: PFGE, MLST, and WGS. The study also evaluated the resistance gene profiles of the isolates. Interestingly, all methods clearly indicated the spread of three different *A*. *baumannii* strains [75]. In our study, the resistance profile of nearly all isolates showed resistance to gentamicin, carbapenems, ciprofloxacin, and tobramycin, which accounted for approximately 92% of all isolates. Similarly, high resistance percentages were observed for amikacin and trimethoprim with sulfamethoxazole (around 83% and 88%, respectively). Among all isolates, we randomly selected 11 CRAB and characterized them molecularly using MLST. In particular, the most common sequence type was ST/281, which has already been previously isolated in other parts of the world [76]. Further studies are therefore necessary to understand the relationship between different STs and AMR. 

Overall, our study suggests that *A*. *baumannii* may have spread across different hospital wards and exhibited a high rate of AMR. This finding is in line with previous studies that have shown infected and colonized patients to be a significant reservoir for horizontal transmission and the spread of *A*. *baumannii* in all healthcare settings [36,37]. A well-recognized fact is that inadequate implementation of IPC measures fosters cross-contamination, leading to a heightened prevalence of *A*. *baumannii* infections in healthcare environments [77,78]. This has been significantly exacerbated by the pandemic, as healthcare facilities and personnel have been overwhelmed by the increasing pressure caused by the admission of COVID-19 patients [28,44]. In addition, our findings confirm the high rate of resistance of *A*. *baumannii*, as previously reported at European, national, and regional levels [4,15,16,23,24,25,79,80,81,82,83]. Several studies have examined strategies to control outbreaks of CRAB during the COVID-19 pandemic. For instance, Mangioni and colleagues [84] conducted a study on the management of a CRAB outbreak in a large ICU COVID-19 hub hospital in Italy. They implemented a tailored IPC strategy that enabled them to contain CRAB transmission while avoiding ICU closure during a critical pandemic period. The IPC strategy implemented in the study involved various measures, such as prompt identification and isolation of patients who tested positive for CRAB, grouping of patients with similar conditions, improvement of hand hygiene, and more rigorous cleaning and disinfection of the environment. In another study, Woon and colleagues [85] conducted an epidemiological investigation of a CRAB outbreak in a neonatal intensive care unit (NICU) and identified weaknesses in the existing infection control measures that may have contributed to the outbreak. The authors propose that the implementation of environmental surveillance, healthcare worker education, and enhanced IPC measures can be effective in controlling CRAB outbreaks in NICUs.

Although our study is the first from a region with limited existing evidence, it has some limitations that need to be discussed. Firstly, it is a descriptive study conducted on a small number of COVID-19 patients who also represented a convenience sample. Due to the high demands and pressures experienced during the initial months of the pandemic, it was not possible to obtain additional information and also isolates from patients who were admitted prior to August 2020. For instance, details regarding antibiotic treatment were not available for all patients, and there was also no information about potential resistance to colistin. Moreover, it cannot be entirely ruled out that some strains may not have been isolated; thus, information on clonality and temporal distribution may be incomplete. Therefore, we were not completely certain that we had identified all of the patients colonized or infected with *A*. *baumannii*. For this reason, we were not able to compare the clinical characteristics and outcomes between COVID-19 patients with and without *A*. *baumannii* infection. Secondly, we did not distinguish between colonized and infected patients, which prevented us from testing for any differences between colonization and infection status. This hindered our ability to determine distinct clinical outcomes and to evaluate if colonized and infected patients contributed differently to the spread of *A*. *baumannii*. Thirdly, the interpretation of PFGE profiles was not performed using specific software, but by visual inspection according to Tenover’s criteria [86]. Finally, MLST analysis was performed on a small number of CRAB isolates, randomly selected from the samples in the study.

Despite these limitations, our study suggests some perspectives for the future that confirm the importance of molecular typing, especially in the current scenario. *A*. *baumannii* is a highly diverse and genetically complex bacterial species, with various strains that can cause different types of infections [87]. Molecular typing can provide important epidemiological information, allowing researchers to track the spread of *A*. *baumannii* infections within and between healthcare facilities [87,88,89]. This can help identify sources of infection and prevent further spread [87,88,89]. Conventional MLST and PFGE are effective methods for characterizing bacterial populations and are considered the gold standard for typing. Specifically, molecular typing techniques can help identify specific strains of *A*. *baumannii* that are associated with COVID-19 and track their spread [87,88,89]. From a clinical point of view, some strains of *A*. *baumannii* may be more resistant to antibiotics than others, which can make them more difficult to treat [87]. However, it is important to combine molecular typing with appropriate assessment of the resistance profile to identify whether certain molecular profiles are linked to specific resistance events [87,88,89]. By identifying the strain of *A*. *baumannii* causing the infection, molecular typing can help guide antibiotic therapy, allowing clinicians to choose the most effective treatment options. 

## 4. Materials and Methods

### 4.1. Study Design and Data Collection

We analyzed data and isolates from patients recruited by the EPIRADIOCLINF project, which aims to uncover the main epidemiological, radiological, and clinical factors related to COVID-19 risk and severe prognosis. Since January 2020, the project has been conducted at the Azienda Ospedaliero Universitaria “G. Rodolico-San Marco” of Catania (Sicily, Italy) and has so far included more than 1200 patients. The study population includes all patients to the ICU, Infectious and Tropical Diseases, and Emergency wards with laboratory-confirmed COVID-19 infection. Data are systematically collected through a standardized electronic form, including patient demographics (e.g., age, gender, residential address, etc.), information on hospitalization (e.g., patient origin, dates of admission and discharge date, status at discharge, etc.), symptoms (e.g., fever, cough, headache, myalgia, nausea, vomiting, diarrhea, etc.); pre-existing comorbidities (e.g., hypertension, diabetes, heart failure, cancer, autoimmune diseases, etc.), risk factors (e.g., smoking habit, obesity, etc.), clinical parameters (e.g., hemoglobin, white cell count, platelet count, creatinine, inflammatory markers, etc.), radiological images, exposure to invasive procedures (e.g., mechanical ventilation, orotracheal intubation, etc.), and drug therapy (e.g., antibiotics, antivirals, monoclonal antibodies, etc.). 

### 4.2. Definition of Carriage, Colonization and Infection Status

*A*. *baumannii* strains isolated from patients included in the study were classified as associated with carriage or colonization/infection. Specifically, the isolation of *A*. *baumannii* in surveillance cultures in hospitalized patients was considered a carriage episode. Colonization is instead defined as the presence of *A*. *baumannii* in a clinical specimen without the presence of clinical criteria that can validate a case of infection [90]. Since it was not possible to validate infections according to clinical criteria, we considered isolates as colonization/infection.

### 4.3. Antimicrobial Susceptibility Testing

In the current analysis, we included *A*. *baumannii* isolates collected from August 2020 to April 2021. Identification and antimicrobial susceptibility testing of *A*. *baumannii* strains were performed using an automatized Vitek 2 system (Biomerieux, Marcy-l’Étoile, France). Antimicrobial susceptibility testing included the resistance to gentamicin (minimum inhibitory concentration, MIC > 4 μg/mL), carbapenems (meropenem and imipenem, MIC > 8 μg/mL), ciprofloxacin (MIC > 1 μg/mL), tobramycin (MIC > 4 μg/mL), amikacin (MIC > 16 μg/mL), and trimethoprim with sulfamethoxazole (MIC > 4/76 μg/mL).

### 4.4. Molecular Typing

*A. baumannii* isolates for molecular typing were isolated as pure cultures and stored at −80 °C with glycerol until analysis. PFGE of the digested genomic DNA was performed to investigate the clonal relationships of the *A. baumannii* isolates. In brief, pure isolates were grown overnight on Muller–Hinton Agar (37 °C overnight). Equal amounts of bacterial suspension, represented by an optical density at 2 McF (6 × 10^8^ cell/mL) in 1× SE buffer (75 mM NaCl, 25 mM EDTA [pH 7.4]) were added to 2% low-meting-point agarose and mixed to form plugs. The bacteria were lysed within the plugs with a cell lysis buffer (0.5 M EDTA [pH 9.5], 1% Sarcosine, 0.015 g of proteinase K per mL) and incubated overnight at 56 °C. This was followed by 5 washes in 5 mL of TE buffer (10 mM Tris-HCl [pH 8], 10 mM EDTA) for 1 h with gentle agitation, repeated for two consecutive days. Next, genomic DNA was digested with an ApaI restriction enzyme, and the macro-restriction fragments were separated using a CHEF-DR III system (Bio-Rad, Hemel Hempstead, UK) at 6 V/cm^2^ for 20 h at 12 °C, and the pulse time was changed from 5 to 13 s. After that, the gels were stained with GelRed Nucleid Acid Gel Stain, and the DNA bands were visualized and photographed under a UV transilluminator. Interpretation of DNA restriction patterns was performed by visual inspection and based on the criteria proposed by Tenover et al. [86]. Isolates that have the same numbers of bands with the same apparent size are designated genetically indistinguishable. Strains showing one to three fragment differences are considered to represent PFGE pattern subtypes. An isolate showing differences in two or three bands is considered closely related to the outbreak strain. Strains showing four to six fragment differences are considered to be possibly related. Isolates are considered unrelated to the outbreak strain if there are seven or more band differences. 

Additionally, we randomly selected one CRAB isolate for each different PFGE pattern (including the subtypes: B1, B2, B3, and B4) for molecular typing using MLST. MLST uses sequences of internal fragments typically of seven housekeeping genes. Specifically, a genetic region of approximately 450–500 bp is sequenced and assigned an allele number that is unique within a species. Each isolate is then characterized by alleles at each of the seven loci that make up its allelic profile or sequence type (ST). For genotyping, we performed MLST on the assembled *A*. *baumannii* genome using the Oxford scheme. The Oxford method was developed and evaluated by Bartual et al. [91]. This scheme contains 2078 different sequence types (ST1 to ST2078, last updated 1 January 2020). In particular, it is designed to identify the following seven internal housekeeping genes: citrate synthase (gltA), DNA gyrase subunit B (gyrB), glucose dehydrogenase B (gdhB), homologous recombination factor (recA), 60-kDa chaperonin (cpn60), glucose-6-phosphate isomerase (gpi), and RNA polymerase sigma factor (rpoD).

### 4.5. Statistical Analysis

Descriptive statistics were used to summarize the main characteristics of the study population and isolates. In particular, we used mean, median, standard deviation, and interquartile range for quantitative variables, or frequency and proportion for qualitative variables. All the statistical analyses were performed using the SPSS software (version 26.0, SPSS, Chicago, IL, USA).

## 5. Conclusions

Overall, molecular typing and resistance profiling of *A*. *baumannii* played important roles in the management of infections during the COVID-19 pandemic. These strategies can aid in the detection and containment of outbreaks, monitoring the development of resistance mechanisms, and facilitating informed treatment decisions to enhance patient outcomes. Our results emphasize the importance of these approaches in a region where such investigations had not previously been carried out. Our study revealed a predominant PFGE pattern that was found in the majority of isolates and COVID-19 patients, along with two subtypes that were detected across multiple wards and over an extended period of time. These findings provide important insights into the transmission dynamics of the disease within the hospital setting. Results from the resistance profiling, instead, showed high percentages of resistance to gentamicin, carbapenems, ciprofloxacin, tobramycin, amikacin, and trimethoprim with sulfamethoxazole. This suggested a concerning level of MDR among the isolates, which may pose a significant challenge for effective treatment of infections caused by these microorganisms. Thus, to enhance IPC practices, as well as adherence to hygiene guidelines among healthcare workers and promote antimicrobial stewardship, it is imperative to implement evidence-based interventions. Furthermore, additional research is required to establish molecular typing as a routine practice for generating crucial epidemiological and clinical insights.

## Figures and Tables

**Table 1:** Antimicrobial treatment in patients with at least one antimicrobial administered.

ATC Class	N.	%
Combinations of penicillins, including beta-lactamase inhibitors J01CR	21	52.5
Third-generation cephalosporins J01DD	7	17.5
Macrolides J01FA	4	10.0
Other antibacterials J01XX	2	5.0
Polymyxins J01XB	2	5.0
Carbapenems J01DH	2	5.0
Glycopeptides J01XA	1	2.5
Tetracyclines J01A	1	2.5
Total	40	100

**Table 2:** Antimicrobial treatment in patients with at least two antimicrobials administered.

ATC Class	N.	%
Carbapenems J01DH	8	22.2
Combinations of penicillins, including beta-lactamase inhibitors J01CR	7	19.4
Third-generation cephalosporins J01DD	5	13.9
Tetracyclines J01A	4	11.1
Fluoroquinolones J01MA	3	8.3
Other antibacterials J01XX	3	8.3
Macrolides J01FA	2	5.6
Polymyxins J01XB	2	5.6
Glycopeptides J01XA	1	2.8
Imidazole derivatives J01XD	1	2.8
Total	36	100

**Table 3:** Antimicrobial treatment in patients with at least three antimicrobials administered.

ATC Class	N.	%
Other antibacterials J01XX	8	30.8
Carbapenems J01DH	5	19.2
Combinations of penicillins, including beta-lactamase inhibitors J01CR	3	11.5
Glycopeptides J01XA	3	11.5
Polymyxins J01XB	3	11.5
Macrolides J01FA	2	4.2
Fluoroquinolones J01MA	1	2.1
Tetracyclines J01A	1	2.1
Total	26	100

**Table 4:** Multi-locus sequence typing (MLST) analysis of eleven isolates with unique PFGE patterns.

Strain Number	PFGE	ST	Allele Number						
			gltA	gyrB	gdhB	recA	cpn60	gpi	rpoD
1	A	238	1	3	3	2	38	97	3
2	B1	281	1	17	3	2	2	99	3
3	B2	281	1	17	3	2	2	99	3
4	B3	281	1	17	3	2	2	99	3
5	B4	281	1	17	3	2	2	99	3
6	C	281	1	17	3	2	2	99	3
7	D	281	1	17	3	2	2	99	3
8	E	238	1	3	3	2	38	97	3
9	F	978	1	17	3	77	2	99	3
10	G	782	56	104	137	67	55	165	75
11	H	1893	1	3	3	77	2	96	3

## Data Availability

The data presented in this study are available on request from the corresponding author.
